# A parapagus dicephalus tripus tribrachius conjoined twin with a unique morphological pattern: a case report

**DOI:** 10.1186/s13256-020-02501-x

**Published:** 2020-10-03

**Authors:** Jeske F. M. Bovendeert, Rutger A. J. Nievelstein, Ronald L. A. W. Bleys, Cindy G. J. Cleypool

**Affiliations:** 1 Department of Pediatrics, Diakonessenhuis, Utrecht, The Netherlands; 2 Department of Radiology & Nuclear Medicine, Wilhelmina Children’s Hospital, University Medical Center Utrecht, Utrecht, The Netherlands; 3 Department of Anatomy, Division of Surgical Specialties, University Medical Center Utrecht, Utrecht University, Utrecht, The Netherlands

**Keywords:** Conjoined twin, Parapagus, Anencephaly, Teratology, Tripus, Tribrachius

## Abstract

**Background:**

Conjoined twinning is a rare congenital malformation with an incidence of about 1.5 per 100,000 births. Because no consensus has been reached regarding the dysmorphology, thorough descriptions of conjoined twins as part of teratological collections can be useful to increase knowledge of this congenital malformation. In this case report, we describe a parapagus dicephalus twin from the collection of the Department of Anatomy of the University Medical Center Utrecht in the Netherlands. External anatomical characteristics were assessed through a detailed macroscopic examination and internal characteristics by means of whole-body computed tomography and magnetic resonance imaging (3 Tesla).

**Case presentation:**

Macroscopic examination showed a Caucasian male parapagus dicephalus tripus tribrachius conjoined twin a type of conjoined twinning in which there are two heads side by side, one rump, and three upper and three lower limbs. In addition, anencephaly was observed in the left twin. Radiological imaging showed a normal central nervous system in the right twin and absence of the calvaria, cerebrum, diencephalon, and mesencephalon in the left twin. There was clear duplication of the vertebral column, rib cage, respiratory system, and gastrointestinal system at least up to and including the first part of the duodenum. The heart consisted of a monoatrium with two separate ventricles. There was a fused liver with a single gallbladder, a single spleen, three kidneys, two bladders, and duplication of the penis. The third upper and lower extremities were articulating with a fused glenoid and acetabulum, respectively. The third foot showed both polydactyly and syndactyly of the toes.

**Conclusion:**

This case report describes a unique case of a male dicephalus parapagus tripus tribrachus conjoined twin discordant for anencephaly. Radiological imaging proved to be an adequate noninvasive method to provide insights into the internal (dys)morphology of this specific specimen, improving its scientific and educational value. This approach could be generally applied to other teratological specimens, thereby strengthening arguments regarding pathogenetic hypotheses, which may lead to new or improved insights into both normal and abnormal embryonic development.

## Background

Many anatomy and pathology departments possess valuable teratological specimens. However, due to lack of detailed external and internal (dys)morphological descriptions and proper diagnoses, these collections are sometimes stigmatized as “morbid cabinets” and not always used to their full scientific and educational potential [1]. Conjoined twins are often found among these collections. Conjoined twinning is a rare congenital malformation with an incidence of about 1.5 per 100,000 births [2] that can be classified according to the most prominent site of union followed by the Greek postfix “-pagus,” meaning “fixed” [3]. No consensus has been reached regarding the dysmorphogenesis of conjoined twinning, which shows there is still much to learn about this congenital malformation [4, 5].

The objective of this case report is to provide a detailed description of both external and internal properties of a unique case of a parapagus dicephalus tripus tribrachus male conjoined twin discordant for anencephaly. These findings will increase the scientific and educational value of this unique teratological specimen.

## Case presentation

The parapagus dicephalus specimen described in this report is part of the collection of the Department of Anatomy of the University Medical Center Utrecht in the Netherlands. The origin, gestational age, and original fixation and preservation method of the specimen are unknown. The specimen is currently stored in a plastic container with a preservation fluid composed of phenol, ethanol, and glycerol (0.2%, 8.3%, and 16.7% wt/vol, respectively). Its external anatomical characteristics were assessed through a detailed macroscopic examination, and its internal characteristics were evaluated by means of whole-body computed tomography (CT) (iQon CT scanner; Philips, Eindhoven, the Netherlands) and magnetic resonance imaging (MRI) (3 Tesla; Philips). Fixed anatomical specimens have tissue characteristics different from those of live tissues, and scanning protocols were optimized accordingly. Tables 1 and 2 list the MRI and CT scanning protocols, respectively. Prior to scanning, the specimen was removed from its plastic container and placed in a disposable plastic bag to prevent dehydration. CT and MRI scans were assessed by a pediatric radiologist specialized in congenital anomalies. A pediatric urologist was consulted to macroscopically assess the genitalia.

**Table 1:** Scanning protocol for Philips Ingenia 3-Tesla magnetic resonance imaging system

Sequence	Voxels (mm^**3**^)	FOV (mm)	TE (milliseconds)	TR (milliseconds)	NSA	TA (minutes)
T1w TFE HR 3D	0.8 × 0.8 × 0.8	128–400	4.61	8.3	2	14.30
T2w TSE 3D	0.8 × 0.8 × 0.8	128–400	90	3000	1	8.54

*Abbreviations: FOV* field of view, *NSA* number of signals averaged, *T1w* T1-weighted, *T2w* T2-weighted, *TA* total acquisition time, *TE* echo time, *TR* repetition time, *TSE* turbo spin echo

**Table 2:** Scanning protocol for Philips iQon computed tomography scanner

Potential (kV)	Current (mA)	ΔSlice (mm)	Increment	Collimation
120	200	0.9	0.7	64 × 0.625

### External characteristics

Macroscopic examination showed a Caucasian male parapagus dicephalus tripus tribrachius conjoined twin, a form of conjoined twinning in which there are two heads side by side, one rump, and three upper and three lower limbs.

#### Head and facial aspects

**Fig. 1:**
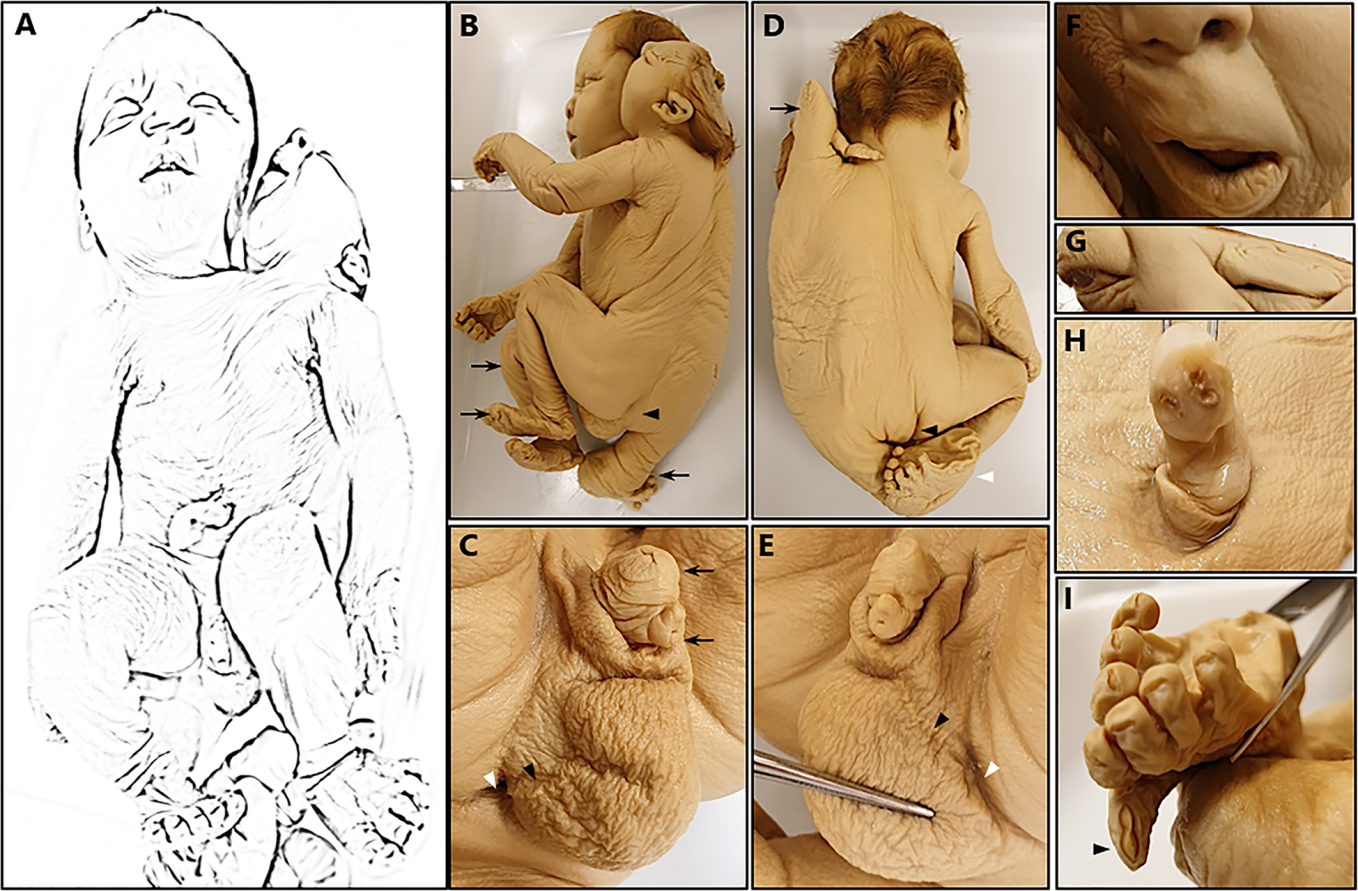
External characteristics of the parapagus dicephalus. **a** Ventral view showing two heads, with anencephaly in the left twin, a shared thorax with a single umbilical cord, and two normal upper and lower extremities. **b** Lateral overview showing oblique positioning of the heads. The gluteal cleft of the left twin (*black arrowhead*) and the three lower extremities (*black arrow*) are distinguishable. **c** Detailed right-sided view of the genitalia showing two penises (*black arrow*), of which the lower-positioned one is hypoplastic. The right perineal raphe (*black arrowhead*) can be followed toward the anal opening (*white arrowhead*). **d** Dorsal overview showing vertical ridge in the midline, the third upper extremity positioned between the heads (*black arrow*), the gluteal cleft of the right twin (*black arrowhead*), and the third lower extremity with a dysmorphic foot (*white arrowhead*). **e** Detailed view of the male genitalia from the left side showing two penises and the left perineal raphe (*black arrowhead*), which can be followed toward a small dimple (*white arrowhead*). **f** Detailed view of the face of the left twin showing flattening of the face and thickening of the philtrum. **g** Detailed view of the face of the left twin showing wide inner and outer canthal distance. **h** Detailed view of the umbilical cord showing three vessels. **i** Detailed view of the dysmorphic foot of the third middle lower extremity with eight discrete digits, of which a single bigger toe with a split toenail (*black arrowhead*) is seen

The right twin’s head and face were normally developed. The slight deformation of the nose was most likely the result of compression during fixation and preservation (Fig. 1a). The left twin’s face was flattened on the right side and positioned obliquely behind the right twin’s head (Fig. 1a, b, and f). The left twin showed anencephaly (Fig. 1b), wide intercanthal and outer canthal distance (Table 3 and Fig. 1g), and thickening of the philtrum (Fig. 1f). All facial abnormalities in this twin could be the result of compression during fixation and preservation.

**Table 3:** Body parameters and corresponding gestational ages for right and left twins

Macroscopic examination	Measured value (mm)		Gestational age (weeks)	
	Right twin	Left twin	Right twin	Left twin
Crown–rump length	285	NA	30	NA
Crown–heel length	430	NA	32	NA
Head circumference	300	NA	32	NA
Outer canthal distance	60	65	35	40
Inner canthal distance	20	25	35	42
Abdominal circumference^a^	310	310	36	36
Internipple distance^a^	55	310	31	31
Foot length	51	57	27	29
CT and MRI scan				
Femur length^b^	58	58	31	31
Biparietal diameter	77	NA	29	NA

*Abbreviations: CT* computed tomography, *MRI* magnetic resonance imaging, *NA* not applicable

^a^Shared structure in left and right twins

^b^Femur length was measured in right and left normally developed lower extremities

#### Neck and torso

The left twin’s neck was slightly shorter than that of the right twin. All observable hairlines appeared normal (Fig. 1b, d). The ventral part of the rump showed a widened thorax and abdomen (Table 3). Two nipples and a single umbilical cord with three blood vessels could be observed (Fig. 1a, h). The dorsal aspect of the torso showed a median vertical protruding ridge, with an extra upper extremity at the cranial end positioned between the heads (Fig. 1d) and an extra lower extremity at the caudal end (Fig. 1b, d). On each side of the caudal part of this ridge, a buttock could be distinguished (Fig. 1b, d).

#### Extremities

The left and right upper and lower extremities appeared normal (Fig. 1a, b). The middle upper extremity was dysmorphic and consisted of one short segment with a single finger attached to it (Fig. 1d). The middle lower extremity was malformed as well and consisted of a normal-sized upper segment and shorter lower segment. The foot showed seven discrete digits, positioned wedge-shaped toward a single, bigger toe with a partial split toenail (Fig. 1b, d, i).

#### Genital and anal areas

The male genital area showed a large single scrotum with two laterally positioned perineal raphae (Fig. 1a, c, e). At the end of the right-sided raphe, toward the right gluteal cleft, an anal opening was observed (Fig. 1c). At the end of the left-sided raphe, a small dimple could be distinguished (Fig. 1e). Cranial to the scrotum, two penises were observed, one positioned above the other. The cranially positioned penis appeared normal, but the other penis was hypoplastic (Fig. 1c, e). Both penises contained a urethral opening.

#### Body measurements and age estimation

Table 3 shows several body parameters measured to objectify anatomical characteristics and to roughly estimate the conjoined twin’s gestational age. The corresponding gestational ages were derived from biometric charts published by various studies on fetal growth [6–8]. Based on fetal growth parameters that could be reliably determined in the specimen (head circumference, biparietal diameter, and crown–rump and crown–heel lengths of the right twin and femur length of the normally developed leg of each twin), the gestational age of the specimen was estimated to be between 29 and 32 weeks.

### Internal morphology

#### Head and central nervous system

**Fig. 2:**
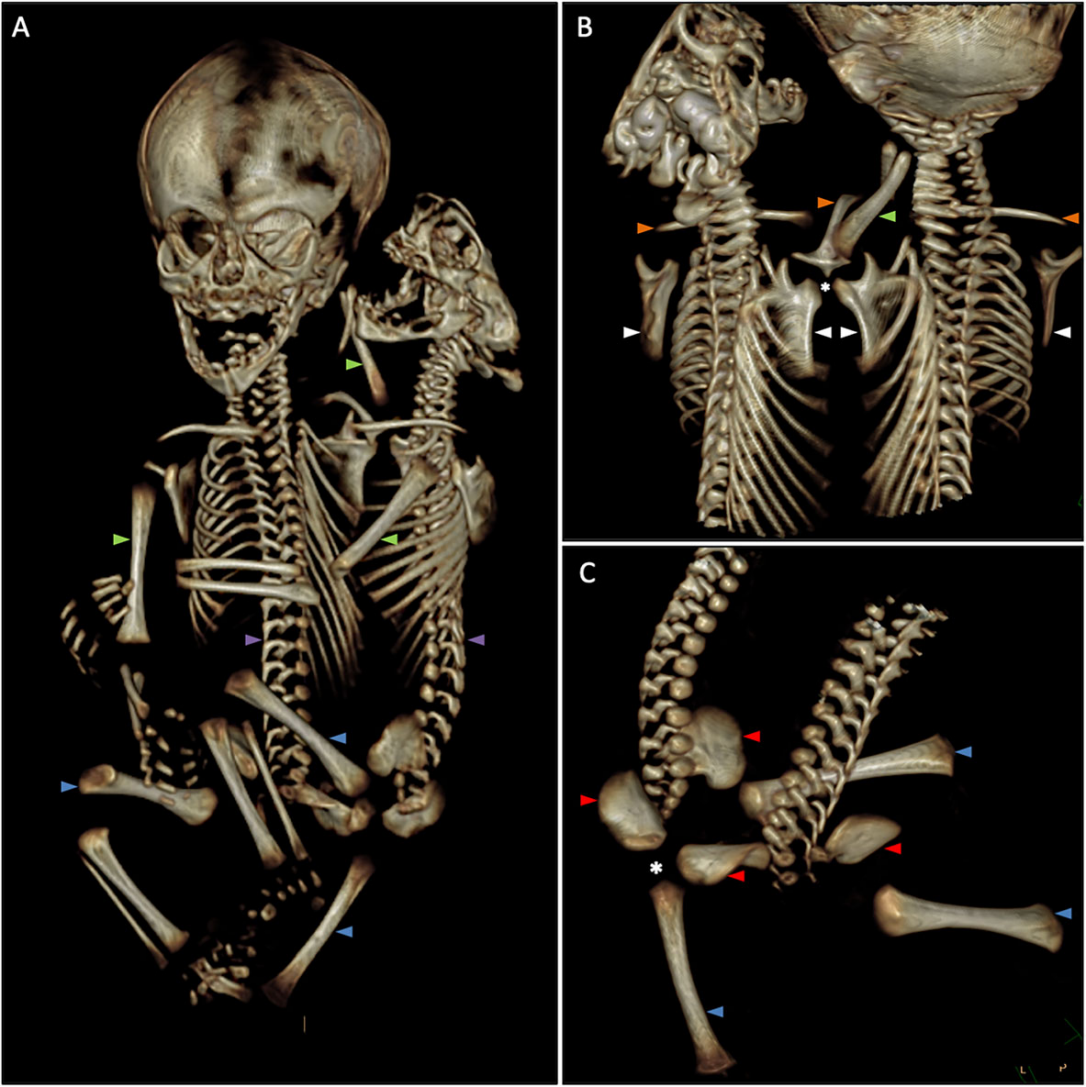
Three-dimensional computed tomographic reconstruction of the skeleton. **a** Overview showing normally developed right twin’s cranium and anencephaly in the left twin. Two separate vertebral columns (*purple arrow*) and three upper (*green arrow*) and three lower extremities (*blue arrow*) can be distinguished. **b** Dorsal view showing details of the third upper extremity (*green arrow*) articulating with a joint (*) formed by the right scapula of the left twin and the left scapula of the right twin (scapulae indicated with *white arrows*). Three clavicular bones can be distinguished (*orange arrow*). **c** Oblique, lateral detail of iliac bones (*red arrow*) and lower extremities (*blue arrow*). The third middle extremity is articulating with a joint (*) composed of the right iliac bone of the left twin and the left iliac bone of the right twin

The right twin’s head showed a normal cranium (Fig. 2a) with well-developed inner ears and a normal brain. A spinal cord was present in the vertebral canal, and the conus was observed at the level of thoracic vertebra T12/lumbar vertebra L1.

In the left twin, the calvaria were absent, and on both sides, not all semicircular canals and cochlear windings could be detected. No cerebrum, diencephalon, or mesencephalon was present, and clear pontine structures were lacking. A clear medulla oblongata could be observed. A small neural structure was seen in the posterior fossa, most likely a rudimentary part of the cerebellum. The vertebral column contained a presumably normally developed spinal cord, but the exact extent could not be determined.

#### Respiratory and cardiovascular systems

**Fig. 3:**
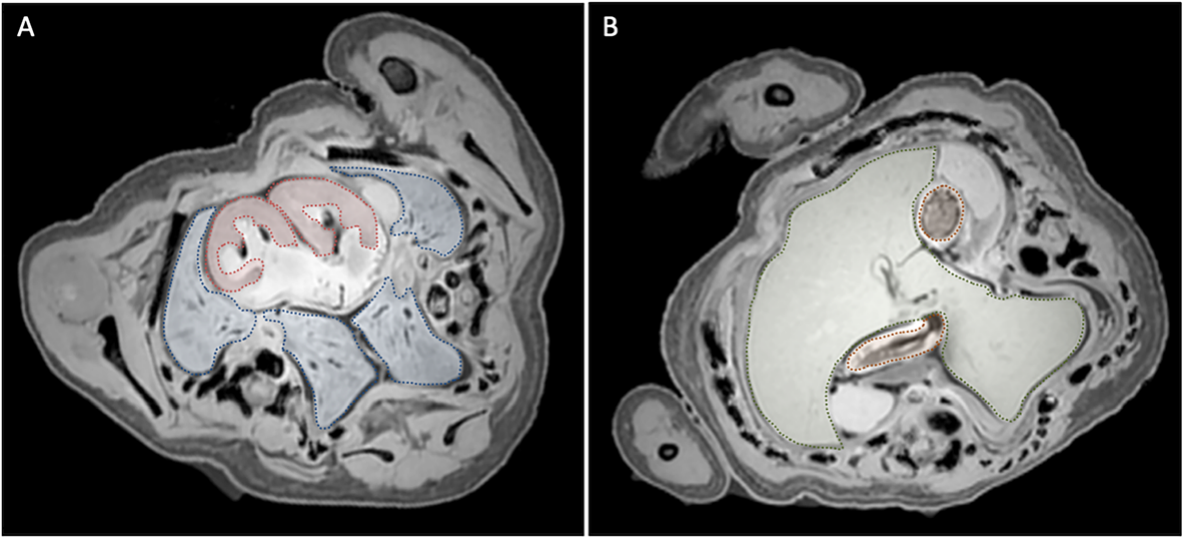
**a** Axial T1-weighted magnetic resonance (MR) image showing a single atrium with two separate ventricles (*red dotted line*) connected and two pairs of lungs (*blue dotted line*). **b** Axial T1-weighted MR image showing a single, fused liver (*green dotted line*) and two stomachs (*brown dotted line*)

Both twins showed normal anatomy of the mouth, nose, and throat, and two separate tracheas could be distinguished. In the right twin, the lowest part of the trachea, the carina, was found at the level of T1, whereas the left twin’s carina was found at the level of T4 (normal being T4/T5). Both twins had their own pair of lungs (Fig. 3a), of which the medially positioned lungs showed no fissures, whereas the laterally positioned lungs showed one oblique fissure each.

The heart consisted of a large single atrium with an auricle on the left side and a possible second auricle orientated more toward the right side. To this atrium, two physically separated ventricles were connected (Fig. 3a). Several vessels drained into the atrium. One central inferior vena cava entered the atrium on the right side at its caudal side. With respect to the thoracic midline, the medially positioned internal jugular veins from both twins merged before draining into the atrium. The right twin’s laterally positioned internal jugular vein drained directly into the atrium, whereas the left twin’s laterally positioned internal jugular vein was not detected. No pulmonary veins could be distinguished.

Each ventricle gave rise to an aorta with an aortic arch. Each arch had three branches toward the neck area. The aorta arising from the left ventricle arched to the left side, and the aorta arising from the right ventricle arched to the right. Both aortae descended on the right ventral side of the corresponding vertebral column.

The umbilical vein passed through one large liver as the ductus venosus, whereafter this structure drained into the single inferior vena cava. Two umbilical arteries were detected, which could each be traced back to an internal iliac artery. One iliac artery was positioned on the right side of the right twin’s pelvis, whereas the other one was positioned on the left side of the left twin’s pelvis.

#### Gastrointestinal system

**Fig. 4:**
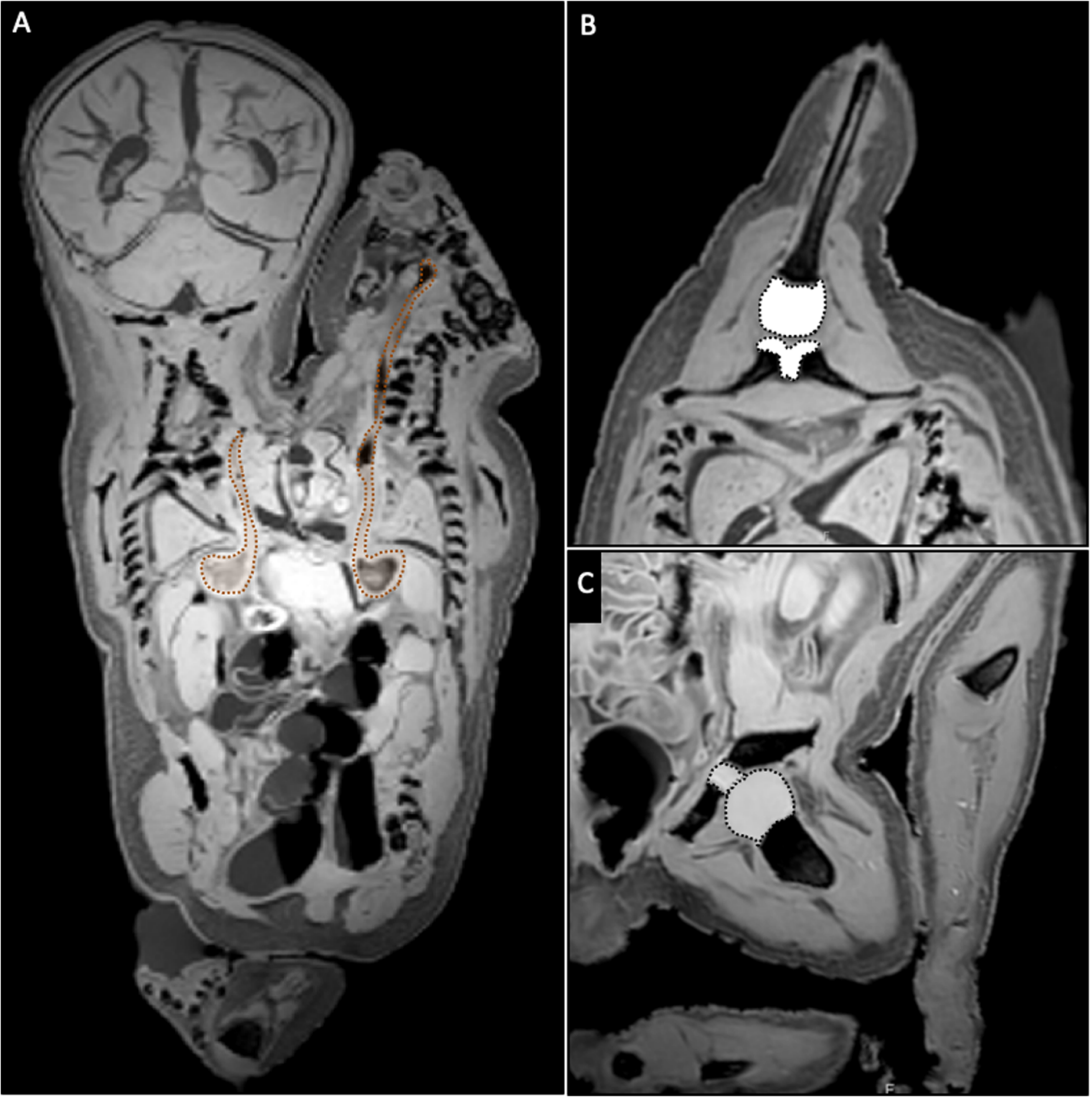
**a** Coronal T1-weighted magnetic resonance (MR) image showing two separate esophagi and mirror-imaged stomachs underneath an overarching diaphragm (*brown dotted line*). **b** Axial T1-weighted MR image showing the hypoplastic bone in the third upper extremity articulating with a cartilage tissue mass positioned between the left and right scapulae of the right and left twins, most likely representing a glenoid. **c** Oblique coronal T1-weighted MR image showing the femoral bone in the third lower extremity articulating with a cartilage tissue mass positioned between the left and right iliac bones of the right and left twins, most likely representing an acetabulum

In both twins, a clear, separate esophagus, stomach, and duodenum could be observed (Figs. 3b and 4b). The abdomen was separated from the thorax by one wide diaphragm. One large liver (Fig. 3b) with a single gallbladder was observed, but no pancreas could be distinguished. A single spleen was positioned on the left side of the upper abdomen. Duplication of the intestine was seen at least up to and including the first part of the duodenum. No statement could be made about the exact extent of duplication of the intestine on the basis of radiological imaging. Only one distal colon was observed, which was connected to a rectum and anal opening on the right side of the scrotum.

#### Genitourinary system

In total, three kidneys were observed. The right twin showed two kidneys with clearly observable, normally positioned adrenal glands. The right kidney showed normal anatomy, whereas the left kidney was dysplastic with multiple small cysts. The left twin showed a left-sided kidney that showed a more elongated shape with the suggestion of two renal sinuses and one adrenal gland on top of it. On the right side of this twin, an adrenal gland could be detected, but the kidney was absent. This combination of findings is suggestive of a renal duplication or crossed, fused renal ectopia in the left twin. One morphologically normal bladder was observed just above and behind the pubic bone. A possible second, smaller bladder was observed more left-sided and toward the dorsal body wall. Both bladders seemed to have their own urethra. No ureters were observed. The small, dorsally oriented bladder narrowed into a small segment cranially, suggesting a ureter stump. At the perineum, a large, fluid-filled single scrotum was observed. No testis could be identified in either the scrotum or the abdominal or pelvic cavity.

#### Skeleton

Two completely separated vertebral columns could be distinguished. There were three clavicles, of which the middle one was shared, positioned between both heads and oriented along the midline (Fig. 2a, b). Two rib cages were distinguished (Fig. 2a). The medially positioned half of each rib cage was positioned more dorsally, and both halves were joined by a “dorsal” cartilaginous sternum. The lateral half of each rib cage was positioned more ventrally, and both halves were attached to a “ventral” cartilaginous sternum. Both twins had their own set of scapulae. The medially positioned scapulae of each twin articulated with the shared clavicle in the midline. The twins both owned a set of iliac bones, but they had only one pubic and one ischial bone each.

The left and right upper extremities showed a normal glenohumeral joint and bone structure. The third upper extremity was composed of one short, hypoplastic bone with one small digit bone attached (Fig. 2b). The epiphyseal cartilage of this extremity attached to articular cartilage positioned between the left and right scapulae of the right and left twins, respectively, most likely representing a fused glenoid fossa (Fig. 4b). The left and right lower extremities were normally developed. The third middle lower extremity was shorter and consisted of a single femur bone and one smaller hypoplastic bone. Its foot clearly showed discrete bones in all eight digits. The epiphyseal cartilage of this extremity appeared to articulate with articular cartilage positioned between the left and right iliac bones of the right and left twins, respectively, most likely representing a fused acetabulum (Fig. 4c).

## Discussion

Several consistent patterns regarding the internal anatomy of different types of conjoined twins have been reported [5, 9]. For parapagus dicephalus twins, this pattern consisted of the presence of two separate brains, variable degrees of duplication of the spine, and a single shared pelvic ring. The location and extent of the shared viscera varied and depended mostly on the number of extremities, the extent of duplication of the vertebral column, the distance between both vertebral columns, and the width of the perineum [9]. Even though many variations have been described [5, 9–15], the morphological pattern of the male parapagus dicephalus tripus tribrachius conjoined twin discordant for anencephaly described in this case report is unique with its almost fully duplicated rump skeleton, heart consisting of a monoatrium with two ventricles, duplication of the penis and bladder, and the third foot showing polydactyly and syndactyly.

There are two main theories regarding the embryonic pathogenesis of conjoined twins: the fusion and fission theory. The fusion theory argues for a secondary fusion between two initially distinct embryonic discs [5]*.* It states that the fusion of two embryonic discs is not random but can only occur at sites where ectoderm is absent or normally destined to fuse or break down during embryonic development (for example, oropharyngeal membrane). The second and most commonly accepted theory, known as the “fission theory,” describes incomplete division of the monozygotic embryonic disc around the primitive streak stage of development (days 13–14 after fertilization) [4]. One of the arguments supporting this theory is the incidence of mirror imaging in up to almost half of the thoracopagus and parapagus dicephalus conjoined twins, most frequently occurring in the right-sided twin [16]. Levin *et al.* [16] studied the cascade of secreted signals during gastrulation regulating left–right asymmetry. They found that when twin primitive streaks form at an angle rather than completely parallel, these signals can interfere between both twins, causing randomization of situs in one of the twins, sometimes resulting in mirror imaging [16].

The specimen presented in this report is most likely the result of incomplete splitting of the embryonic disc. Two parallel primitive streaks, closer at the caudal end and wider apart rostrally, could explain why the rostral region showed a more distinct duplication pattern of organs and structures (head and foregut structures) than in more caudally positioned organs and structures. Further supporting this assumption was the presence of mirror imaging observed in the right twin, best visualized in the position of both stomachs (Fig. 2b) and in the orientation of the aortic arches.

In addition to the unique morphological pattern, the parapagus dicephalus specimen described here showed anencephaly in the left twin. Although not common, two previous case reports described the combination of these congenital anomalies [10, 11]. Both cases concerned parapagus dicephalus dipus dibrachius twins. In the first case, the anencephaly was seen in the right twin, whereas in the second case, the left twin was affected. Any disruption of the dynamic sequential events of neural tube closure (NTC) can cause neural tube defects (NTDs), leading to deformities such as spina bifida, exencephaly, anencephaly, and craniorachischisis in the newborn. The occurrence of NTDs is explained by two theories: (1) abnormal cell behavior (that is, insufficient cell proliferation, dysregulation of cell death, or inappropriate collective cell movement) that results in disrupted NTC or (2) reopening of the tube, a theory also referred to as the “time window hypothesis” [17]. Anencephaly is not the only NTD described in conjoined twins; spina bifida and craniorachischisis are also reported [12–14, 18]. The frequent occurrence of the combination of conjoined twinning and NTDs suggests a common etiological factor contributing to both congenital conditions. To date, no valid hypothesis on a common etiology has been suggested.

This is not the first time radiological imaging was used as a noninvasive method to increase insight into the internal dysmorphology of a teratological fetus. Boer *et al.* used an approach similar to ours, in which 41 teratological specimens underwent both full-body CT and MRI [1]. With the wide availability of prenatal screening, most congenital anomalies are detected early in pregnancy, often resulting in termination [15]. This, in combination with the fact that stillborn fetuses are sparsely donated for scientific purposes, increases the value of scanning and describing teratological full-term fetal specimens of teratological collections. With radiological imaging, the fetus remains intact, and, even after long-term preservation, the radiological data have proved to be sufficient to describe most of the internal characteristics of the fetus. CT and MRI complement each other, with, for instance, skeletal structures best visualized with (three-dimensional) CT and MRI, providing more details on internal viscera and cartilaginous structures.

Significant limitations should be taken into account when working with teratological specimens from anatomical and pathological collections. First, most of these teratological specimens have been preserved long term in containers with formaldehyde solution for multiple years. Mechanical compression– and/or formaldehyde-induced tissue alterations could lead to deformation of external and internal structures, hampering proper morphological description.

Second, although modern imaging techniques are widely used in postmortem investigations, their diagnostic capacity is limited, and, in most cases, traditional autopsy remains indispensable for both diagnosis and determination of the cause of death [19, 20].

### Conclusion

This case report describes a unique case of a Caucasian male dicephalus parapagus tripus tribrachus conjoined twin discordant for anencephaly. Radiological imaging proved to be a successful noninvasive method of increasing insight into the internal (dys)morphology of this specimen, improving its scientific and educational value. This approach could be applied to other teratological specimens, thereby strengthening arguments regarding pathogenetic hypotheses, which may lead to new or improved insight into both normal and abnormal embryonic development.

## Data Availability

Data will be made available if requested.

## Abbreviations

CT: Computed tomography
FOV: Field of view
L: Lumbar vertebra
MRI: Magnetic resonance imaging
NA: Not applicable
NSA: Number of signals averaged
NTC: Neural tube closure
NTD: Neural tube defect
T1w: T1-weighted
T2w: T2-weighted
TA: Total acquisition time
TE: Echo time
T: Thoracic vertebra
TR: Repetition time
TSE: Turbo spin echo
